# A System for Household Enumeration and Re-identification in Densely Populated Slums to Facilitate Community Research, Education, and Advocacy

**DOI:** 10.1371/journal.pone.0093925

**Published:** 2014-04-10

**Authors:** Dana R. Thomson, Shrutika Shitole, Tejal Shitole, Kiran Sawant, Ramnath Subbaraman, David E. Bloom, Anita Patil-Deshmukh

**Affiliations:** 1 Department of Global Health and Social Medicine, Harvard Medical School, Boston, Massachusetts, United States of America; 2 Partners for Urban Knowledge, Action, and Research, Mumbai, India; 3 Division of Infectious Diseases, Brigham and Women’s Hospital, Boston, Massachusetts, United States of America; 4 Department of Global Health and Population, Harvard School of Public Health, Boston Massachusetts, United States of America; Tulane University School of Public Health and Tropical Medicine, United States of America

## Abstract

**Background:**

We devised and implemented an innovative Location-Based Household Coding System (LBHCS) appropriate to a densely populated informal settlement in Mumbai, India.

**Methods and Findings:**

LBHCS codes were designed to double as unique household identifiers and as walking directions; when an entire community is enumerated, LBHCS codes can be used to identify the number of households located per road (or lane) segment. LBHCS was used in community-wide biometric, mental health, diarrheal disease, and water poverty studies. It also facilitated targeted health interventions by a research team of youth from Mumbai, including intensive door-to-door education of residents, targeted follow-up meetings, and a full census. In addition, LBHCS permitted rapid and low-cost preparation of GIS mapping of all households in the slum, and spatial summation and spatial analysis of survey data.

**Conclusion:**

LBHCS was an effective, easy-to-use, affordable approach to household enumeration and re-identification in a densely populated informal settlement where alternative satellite imagery and GPS technologies could not be used.

## Background

Conducting research and outreach in slum communities is challenging for several reasons, including that the absence of a street address system makes it difficult to uniquely identify and follow up with households. Alternative household enumeration methods, including use of GPS units and satellite imagery, do not work in densely populated slums. With one in six people living in slums worldwide, including one out of every three city dwellers [1], household enumeration and identification poses an enormous challenge for researchers, community organizers, and advocates working with the urban poor. Slum environments are also challenging because they change rapidly. Residents move frequently, and structures are rebuilt often due to poor construction, natural disaster, change in tenant configurations, local politics, and forced eviction [2]. In these dynamic settings, enumeration of the community’s residents, physical structures, and boundaries aids in recognition of the community’s existence which can serve as a tool to lobby officials for pro-poor housing policies and participatory slum redevelopment [2]. Adaptive, easy-to-implement housing enumeration methods that can be used by slum dwellers and researchers alike in high-density settings are sorely needed.

Various methods have been used for slum enumeration, including hand-drawn maps on paper or in a geographic information system (GIS) [3], mapping with GPS units [4], enumerating from satellite imagery [3], or some combination of the three [5]. Advanced technologies, such as spatially encoded video [6], have also been used to capture community health risks. Each enumeration method poses challenges. Drawing maps by hand is time-intensive and requires many enumerators. Use of GPS units and spatially encoded video require a technical team to collect, clean, and maintain spatial data, and technically trained workers or volunteers may not be readily available [7]. Members of our team who worked with organizations in other slum settlements found that locating a household with a GPS unit and a printed map (a common way to identify households coded by GPS) requires 20–30 minutes in the field per household to ground-truth the GPS reading and converse with residents in nearby homes. Furthermore, use of GPS is problematic in densely populated slums because current GPS units have a spatial error of up to 33 feet (10 meters), and building cover prevents satellite connection [7], [8]. High building density is especially problematic in a city such as Mumbai, where over half of the population lives in slums on six percent of its land [9], making Mumbai’s slums some of the highest-density settlements on earth. For example, Dharavi, one of Mumbai’s largest slums, is estimated to have a population density greater than 400,000 people per square kilometer, which is nearly ten times denser than daytime Manhattan [10]. Enumeration from recently collected satellite imagery can be very efficient and accurate [11], though in very densely populated slums like Dharavi, structures are stacked several stories high and share walls so that the roofs belonging to different dwellings are indistinguishable.

**Figure 1:**
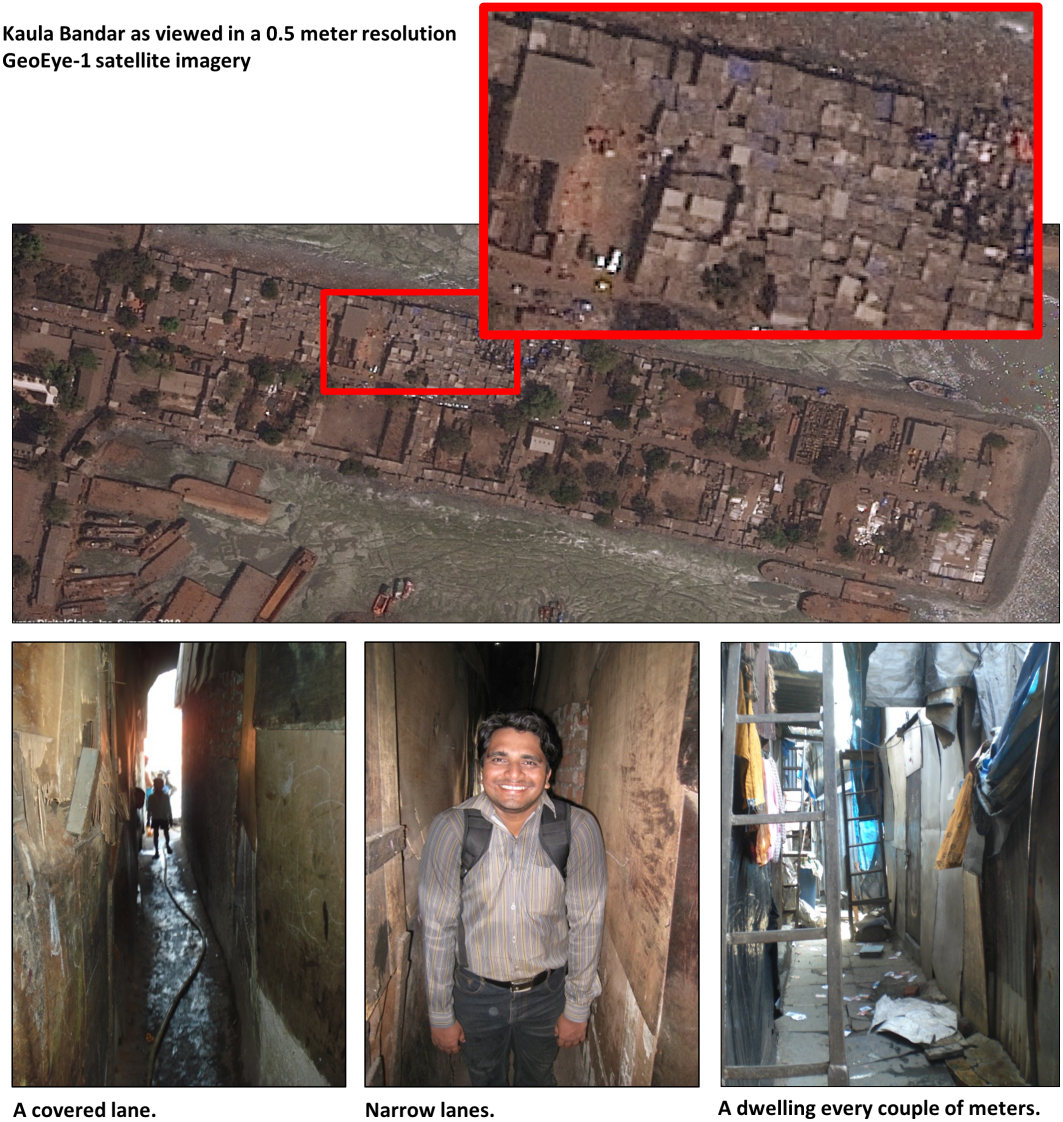
Kaula Bandar, Mumbai, India: A densely populated slum.

**Figure 2:**
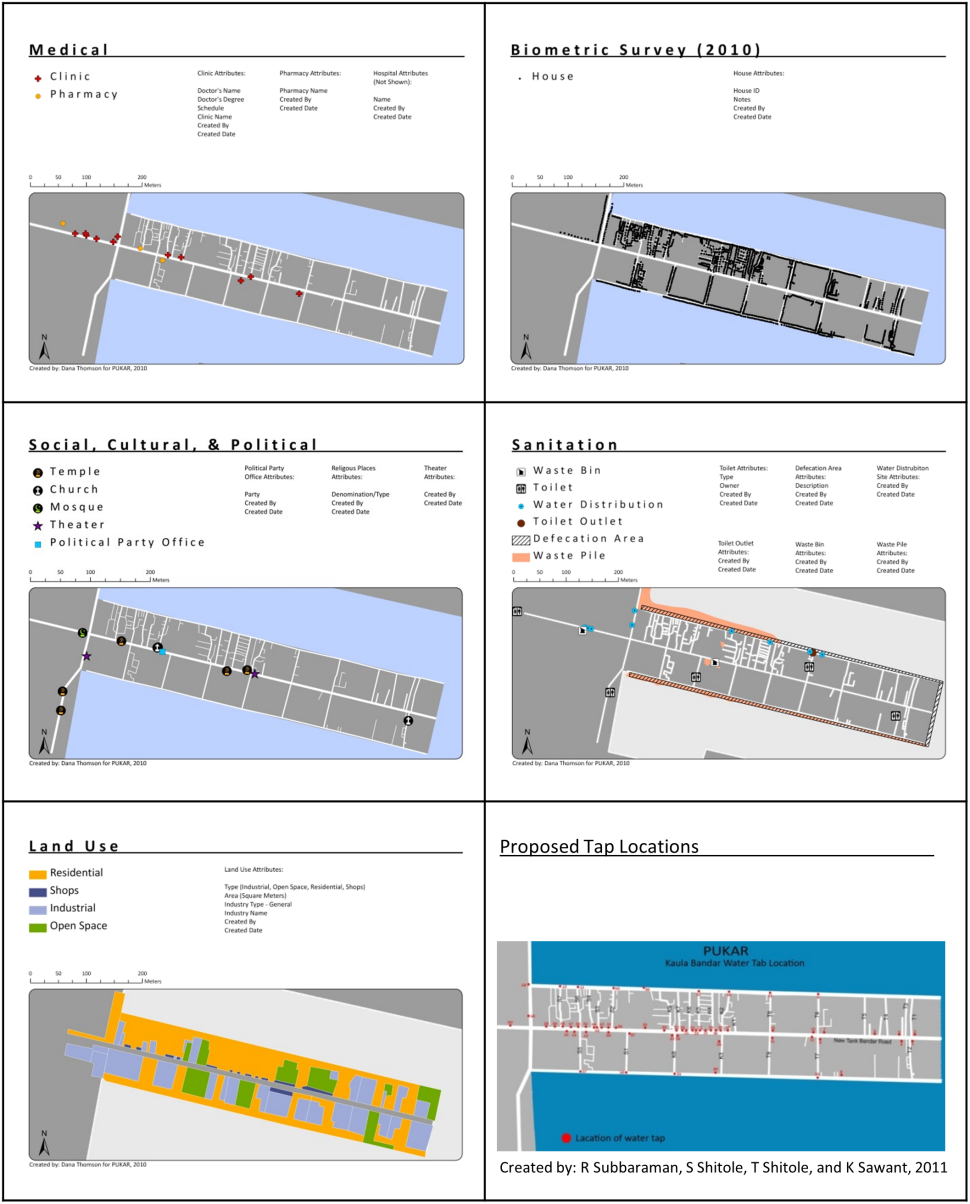
Example community maps.

In 2008, researchers from Partners for Urban Knowledge, Action and Research (PUKAR); Harvard School of Public Health (HSPH); and New York University (NYU) formed a collaboration to perform a series of health studies, and health and education campaigns in Kaula Bandar, a slum of approximately 14,000 people in Mumbai, India. The slum is located on a former shipping pier one-tenth of a square kilometer in size with one road passable by motor vehicle down the center, and a labyrinth of pedestrian-only lanes on either side, most of which are covered by buildings. Many of the lanes are so narrow that individuals can only pass through in a single-file configuration, and larger individuals may need to turn sideways to navigate some of the narrow spaces (Figure 1). Approximately half of the land space in Kaula Bandar is residential - the rest is industrial or open space - which means that residential density is approximately 280,000 people per square kilometer (see Figure 2). Dwellings in Kaula Bandar range in size from 20 to 90 square feet (2 to 8 square meters), and are home to 5 people on average.

Kaula Bandar is a “non-notified” slum, which means it is not recognized by local and state governments. Non-notified status greatly limits residents’ ability to pressure officials for basic water and sanitation infrastructure, schools, or health services [12]. As a result, all water, sanitation, and power infrastructure is improvised by residents except for a few public toilet facilities. PUKAR is unique in that the organization is staffed by a small national research team that hires and trains a cadre of what Arjun Appadurai calls “Barefoot Researchers” from the community to collect data and perform community education and outreach [13]. Barefoot Researchers are often young (ages 18–23), and many have limited formal education. Engagement and training of Barefoot Researchers was conducted in the spirit of what Kretzmann and McKnight call Community Asset Mapping; that is, taking inventory of the capacities, skills, and assets of low-income people and their communities to reinforce community pride and internally generated development, rather than document needs and deficiencies which reinforces dependency on outsiders [14]. In this way, research, education, advocacy, and community building in Kaula Bandar are integrated and informed by one another.

Nearly half of Kaula Bandar’s adult residents have lived in the slum for more than 20 years, and its population continues to grow as new residents move in and young adults start their own families. As a result, new lanes are formed by the construction of new dwellings. Typically, a dwelling’s ground level is occupied by the owner, while 2^nd^-level units are rented. A large portion of Kaula Bandar’s residents move away seasonally for work or to care for relatives in rural villages. The majority of dwellings in Kaula Bandar have existed for years, though we estimate that the Bombay Port Trust demolished at least 5% of homes in the last five years targeting new construction in open spaces and on the main road, and large fires in 2010 and 2013 destroyed approximately 9% and 3% of dwellings, respectively. After these fires, government support to rebuild was promised, though only a few politically connected families received funds, and only after months of delay. Most burned dwellings were rebuilt and reoccupied by the same tenants using private resources, and those households that started rebuilding first slightly increased the size and quality of their dwellings.

In this densely populated community with regular changes to housing and population, none of the typical enumeration methods are feasible; GPS units cannot make satellite or cell phone network contact from narrow, covered lanes, and dwelling rooftops are indistinguishable in satellite imagery. While hand-mapping is an option, it requires extensive human-power and time. In this paper, we describe a method developed by the PUKAR research team to code and find households efficiently, present how we extended the coding system for mapping and spatial analysis, and discuss the utility of this method for research and practice in other densely populated or dynamic slum settings.

## Methods

### Location-based Household Coding

PUKAR’s three lead field researchers (authors SS, TS, and KS), two of whom are from communities near Kaula Bandar, created a simple, innovative Location-Based Household Coding System (LBHCS) to uniquely identify households. This system allowed them and each of the Barefoot Researchers to find their way to a specific household in a matter of minutes. The PUKAR team started with a simple hand-sketched map of Kaula Bandar that included the main road and each of the community’s 25 main lanes connecting the central road with the edge of the pier (see Figure 1). At the time that the system was developed, each of the three researchers led a team of Barefoot Researchers in a section of the community. Main lanes were assigned codes like T4, S3, and K7, indicating it was the 4^th^, 3^rd^, or 7^th^ main lane in Tejal, Shrutika, or Kiran’s section. Each sub-lane was coded with the direction (left or right) from which it originated from the main lane (from the perspective of where the pier connects to land), and the order in which it appeared (1^st^, 2^nd^, 3^rd^ sub-lane). Additional branches of sub-lanes and smaller by-lanes were accordingly coded, resulting in such codes as K9-LSL2-LBL1, which represented the 9^th^ main lane in Kiran’s section, 2^nd^ left sub-lane, 1^st^ left by-lane. Special codes were created for homes near to the sea (NS) and the road outside of Kaula Bandar (RO). Specific home locations were indicating by adding a side of the lane and house number to the lane code, as in K9-LSL2-LBL1-R3D, the 3^rd^ house on the right, downstairs, on lane K9-LSL2-LBL1. Second-story households were coded as U for upstairs level. In this way, household codes doubled as detailed, easy-to-follow walking directions and the basis for a relational database of all addresses at a particular point in time.

For each study, PUKAR maintained a database of each participant and their household code. Each time the participant was visited in their home for data collection, medical follow up, or educational outreach, the field researcher verified the accuracy of the household code, and noted on paper any corrections resulting from previous recording error or because the participant had moved. Updated household codes were entered into in an excel database by the database manager who tracked all old or incorrect codes in a separate column.

### GIS Mapping

As noted, Kaula Bandar is so densely populated that individual households are indistinguishable in satellite imagery. And most lanes, sub-lanes, and by-lanes are covered by housing so they do not receive GPS signals and they are not visible in satellite imagery. PUKAR researchers (authors SS, TS, KS) with extensive knowledge of the community mapped all lanes in a GIS with the help of a GIS student (author DT) using detailed satellite imagery and a few key GPS coordinates for reference. GPS coordinates were taken at the beginning and end of each main lane, and the satellite imagery provided additional cues about lane locations, such as “seams” over lanes formed by rooftops.

After the lane network was mapped, it was straightforward to map all households based on a household census with LBHCS. A household census using the coding system indicated the total number of households located on each side of each lane segment. The GIS student generated a latitude/longitude coordinate for each ground-level dwelling (upstairs dwellings were assigned the same coordinate as downstairs dwellings), which resulted in a map of all addresses. Although the coordinates do not identify dwelling centroids (geographic center of a shape), the resulting spatial data can be thought of as a household entrance, similar to an address geocoding service such as GoogleMaps. The household locations are sufficiently accurate for property identification (for example, locating a claim to rebuild after a fire), spatial analysis (for example, hot spot analysis), calculation of distance variables (for example, distance from home to nearest water tap), and establishing the relative location of neighbors.

Several additional spatial datasets were generated from the satellite imagery by a PUKAR researcher (author KS) and the GIS student (author DT) that were not dependent on LBHCS, but were essential for community organizing and advocacy (Table 1). These spatial datasets included information on basic infrastructure such as toilets, trash bins, trash piles, and water distribution sites, as well as community resources such as temples, religious pre-schools, and informal health clinics and pharmacies.

**Table 1:** Spatial data layers and attributes collected.

#	Spatial Data Layer	[Vector type] Attributes
**Base Layers**		
1	Health “Clinics”	[point], clinic type, clinic name, degree and name of service provider, hours
2	Pharmacies	[point], name
3	Industries	[polygon], name, type
4	Land use	[polygon], land use type, square meter
5	Lanes and Sub-lanes	[line], name
6	Police Stations	[point], name
7	Political Party Office	[point], party name
8	Ration Stations	[point]
9	Religions Places	[point], denomination
10	Religious Pre-Schools	[point], name, type, highest level, funding source
11	Theaters	[point], type
12	Toilet outlets	[point]
13	Trees	[point]
14	Waste bins	[point]
15	Waste piles	[polygons]
16	Water distribution sites	[point]
**Advocacy & Research Layers**		
17	Defecation Areas	[polygon]
18	Health Zones	[polygon], name
19	High Tide Flood Areas	[polygon]
20	Hospital	[point], name, type
21	House Addresses	[point], household id
22	Monsoon Season Flood Areas	[polygon]
23	Proposed Water Distribution Sites	[point]

### Resources

Our tool kit included a 25 square kilometer, 0.5 meter resolution GeoEye-1 satellite image purchased with an academic license for US $230 from a commercial provider (eMap), and a walking census of households using LBHCS. In 2010 and 2012, PUKAR researchers (authors TS, SS, KS) collected unique household codes for all households in the community in just four days, a database manager entered these data within two weeks, and a PUKAR researcher (author KS) and a GIS graduate student (author DT) mapped thousands of features and their attributes directly in a GIS within three weeks. The success of this project hinged on a small team with strong local place knowledge, database management skills, and GIS skills. We used a computer with Excel (a spreadsheet program) and ArcGIS (a geographic information system), which at the time of this writing cost US $1500 for a single ArcGIS license plus a few hundred dollars for a computer with the MS Office Suite. Free spreadsheet and GIS programs such as Apache OpenOffice and QGIS were also available. We borrowed one GPSMAP 76CSx GPS device to collect the geographic coordinates of key landmarks, including the start and end of covered lanes.

**Figure 3:**
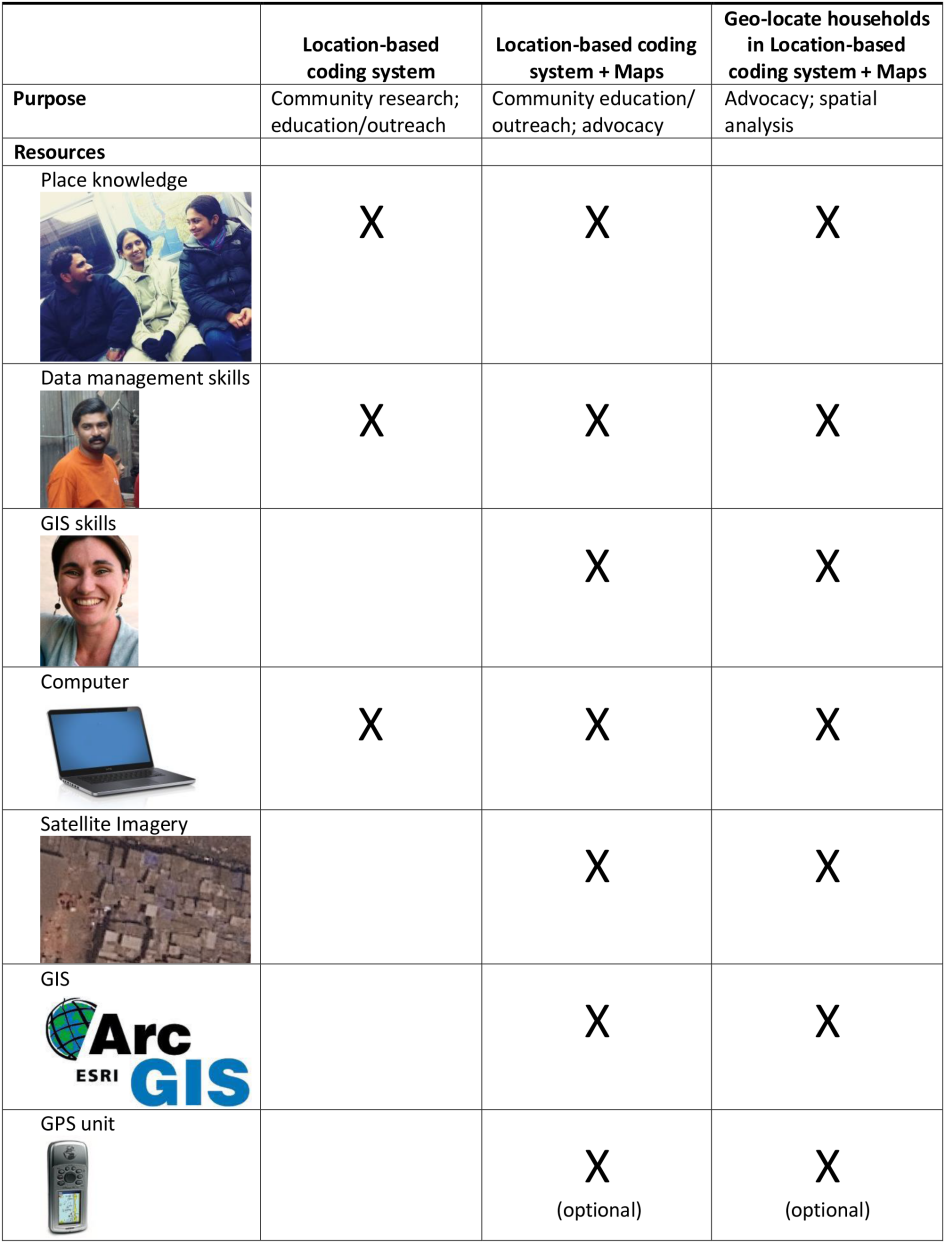
Location-based household coding system toolkit and applications.

LBHCS can be used alone for data collection and outreach by minimally trained staff at low cost. If using the household coding system alone, it is important to have at least one team member who is familiar with data entry and data cleaning, and to have a computer. If advocacy or quantitative research are key activities, we recommend integrating the household coding system with mapping activities as we present here. See Figure 3 for a summary of this toolkit.

### Maintenance of the Data

LBHCS can be used in one of two ways. Users can either carry out a household census at one point in time and update it periodically, or users can regenerate household censuses periodically creating new unique household codes in each census that are not linked to previous censuses. The ideal approach will depend on the community and the reason for using a household coding system. In slums in which structures are knocked down or replaced regularly, it may be advantageous to regenerate household codes periodically so they remain useful as walking directions and capture an accurate snapshot of the community’s residents and layout. In slums with a stable set of structures, it may make sense to maintain and update a longitudinal database of household codes and locations.

If new households are built between existing dwellings, the function of the codes as walking directions decreases; therefore, we recommend recoding households periodically (for example, annually), keeping track of previous household codes and their dates of use. However, for minor additions, users can add half numbers (as in K9-LSL2-LBL1-R*3.5*D) or a letter for new dwellings (as in K9-LSL2-LBL1-R*N*D). Notably, in Kaula Bandar, the pre-existing density of dwellings was very high (with nearly all dwellings being immediately adjacent to other ones), so we rarely ran into situations in which new dwellings could be built in between others. Most new homes could only be built at the very ends of existing lanes or in completely new lanes, which did not interfere with the utility of the coding system. As such, the coding system was very useful for longitudinal follow-up of homes in Kaula Bandar; however, in less dense settlements, this may not be the case.

Maintaining a longitudinal dataset of household IDs requires substantially more data infrastructure and skills than simple cross-sectional uses of the coding system, and may not be worthwhile for advocacy, education, or research teams that operate on shorter time scales. This observation applies to, for example, education programs staffed by students, or researchers working on shorter grant cycles. In those slums in which household IDs are not maintained, the IDs may still facilitate follow-up for many months, which is often the duration of an intervention, educational campaign, or study.

### Ethics Statement

A number of studies were conducted in Kaula Bandar using LBHCS including a biometric census, longitudinal water quality study, cost of diarrheal illness study, and mental health study. The biometric census included adults and children, while the other three studies included only adult participants. Residents were willing to participate in these studies largely because of PUKAR’s community-based participatory research model, which involved youth from the Kaula Bandar community in data collection. In addition, findings were used to directly advocate for improved outreach to residents by the local government.

In the biometrics study, signed consent, or thumbprint consent for individuals who could not or did not know how to spell their names, was obtained from all adults and guardians of minors. During the study, the PUKAR team received feedback that many participants were hesitant to sign or provide thumbprints, even if they wanted to participate in the study, as these actions are associated with government or police interactions. As a result, verbal consent was obtained for all subsequent studies with the signature of the researcher. For interviews that required audio recording, the participant’s consent was also digitally recorded. All study protocols, including the consent forms, received approval from either the Institutional Review Board of the Harvard School of Public Health (17740) or from the PUKAR Institutional Ethics Committee (FWA00016911).

## Results

### A Household Coding System for Community Research and Outreach

From March to December 2010, the PUKAR team engaged in an extensive biometrics survey in Kaula Bandar, in which basic demographic and health information, height, weight, blood pressure, and a photo (holding a chalkboard with the individual’s household ID) were collected from community residents at a central research station that shifted to different parts of the community on the main road. All households in Kaula Bandar were coded by PUKAR researchers immediately prior to starting the survey. Approximately 1200 new residents moved to Kaula Bandar during the 10 months of the study. Every weekend, Barefoot Researchers were provided with a list of household codes. With minimal training about the coding system, the Barefoot Researchers were able to (1) independently identify each household in the community, (2) perform a household census, (3) recruit individuals to the main research station, and (4) perform home-based follow-up with nearly 100 individuals who had hypertension at the research station and whose blood pressure was re-measured so the individual could be referred to the hospital for appropriate evaluation, if needed. Moreover, each study subject’s health information and photo were printed on a card, and these health cards were returned to residents to provide them with a basic health record. Using the coding system, Barefoot Researchers returned roughly 96% of health cards to residents within a week of initial data collection, as repeat identification of homes was easily and quickly performed by any of the research team members.

In 2011, PUKAR performed a study of water issues in the community, in which a consistent set of 21 homes located throughout the slum were followed longitudinally for a year to assess water cost, quantity, and quality, using serial microbiological testing of water samples [12]. On days when water testing was performed, it was important to locate all homes very rapidly, as water samples had to be transported in bulk to the laboratory within a couple of hours of collection. Without the household coding system, such rapid repeat identification of homes throughout the community on the same day would not have been feasible. The coding system also showed remarkable stability over a year of follow-up, largely because the community is so dense that new homes cannot be constructed between pre-existing homes.

In 2012, PUKAR engaged in a study of mental health in Kaula Bandar, for which a random sample of households was required. Since the previous coding of community households in 2010, a few new lanes had been added to the community, and new households had been added to the ends of lanes. As such, we decided to re-code the entire community to ensure an accurate roster for randomized sampling. With a team of ten people, all households in the settlement were re-coded in less than four days. This new registry allowed us to select a subset of households for the mental health survey using a random number generator.

In 2011 and 2012, the PUKAR team engaged in extensive door-to-door education of community residents on critical health topics such as child immunizations and diarrheal disease. The household coding system allowed Barefoot Researchers to record homes that were missed in initial rounds of the educational efforts, such that repeat efforts could be made to find the residents of these households to make sure they received individualized education.

### Maps for Advocacy and Outreach

The PUKAR research team worked with a GIS student to map 23 spatial layers of existing infrastructure and services, and produce an atlas of maps that the team has since used extensively for community outreach and advocacy (Figure 2). In October 2010, for example, PUKAR was invited to present its work to Mumbai’s Municipal Health Commissioner as part of a consultation of the city government with non-governmental organizations (NGOs). In preparation for this presentation, PUKAR researchers mapped nearby government health centers to emphasize the distance of Kaula Bandar from these resources. These maps graphically highlighted the community’s isolation from public health centers, and they were critical in motivating the municipality of Mumbai to extend twice-monthly health camps to Kaula Bandar for provision of immunizations and other basic medical care. These efforts, along with door-to-door education efforts by PUKAR, helped to improve the fraction of children who are fully immunized with the basic set of vaccinations in Kaula Bandar from 29% to 80% in just three years [Unpublished results presented by PUKAR at the 2012 NIH Summit on the Science of Eliminating Health Disparities].

After PUKAR completed a study on water poverty in Kaula Bandar [15], PUKAR researchers presented their data to Mumbai’s Municipal Commissioner for Water in late 2011, including a map of Kaula Bandar’s limited water and sanitation infrastructure. As a result of this dialogue, the Water Commissioner took steps to facilitate extension of a new water supply to the Kaula Bandar community. The development of a new water supply is still in process, as fundamental water infrastructure needs to be extended to the entire nearby area before the local water supply in Kaula Bandar can be developed further. The Water Commissioner and the local corporator (equivalent to an alderman) suggested that once this main supply is in place, approximately 60 new community water taps can be placed in Kaula Bandar.

PUKAR researchers have concerns regarding equitable placement of these water taps, as studies have shown that the distance of a household from a water source may have a major impact on health outcomes [16]–[18]. Given these concerns, PUKAR researchers proposed a plan for community water tap placement and mapped it (Figure 2). Tap placement on the map was decided based on the population density in each lane, as well as PUKAR researchers’ knowledge about open spaces in the slum to facilitate ease of access and mobility within the community. PUKAR presented this plan to the Water Commissioner and local corporator, and both officials agreed on this plan for tap placement. We hope that this map remains a shared roadmap for the community and government officials, and facilitates government accountability for equitable water tap placement.

### Spatial Data for Research and Advocacy

The research team at Harvard used the household coordinates to reaggregate data from a biometric study to sub-community health zones to perform spatial summaries and analyses of exposures and outcomes. Health zone boundaries corresponded to landmarks. The team distinguished households located near the center or at the periphery of the pier because households on the periphery were believed to have higher exposure to contaminated water due to high tide and monsoon flooding. As noted, Kaula Bandar has a small geographic footprint, covering less than one-tenth of a square kilometer of land. We found little spatial variability of health outcomes and exposures within the slum; all of our exploratory spatial analyses produced null results. In larger slums with greater variability in terrain, resources, or within-slum socioeconomic disparities, use of the household coding system with geolocated addresses would facilitate extensive spatial analysis including spatial regression and cluster analysis (also called “hot spot analysis”).

### Maintaining a Household Coding System Over Time

The coding system worked well with the fluid, malleable nature of the community. Kaula Bandar is fluid in two ways. First, people in the community migrate seasonally to rural areas and residents who accrue resources migrate to “better” slums. They also move because of demolition of their homes by the government and eviction by owners of the dwelling. The dwellings also change periodically. Homes disappear and reappear due to fire, and some of the homes on the main road get periodically demolished. As such, the coding system allowed us to re-enumerate the community in four days and account for changes in living structures due to expansion, fires, and demolition. By integrating LBHCS with spatial data collection we were able to keep a permanent record of former household locations and lane configurations.

## Discussion

Urban slums comprise one-sixth of the global population [1], but can be difficult to work in or study because lack of an address system limits follow-up with households over time. Household enumeration systems are needed in slum communities that comprise a substantial portion of the global population, particularly in high-density slums in which GPS units, satellite imagery, and other common enumeration tools do not work. We created an innovative Location-Based Household Coding System that facilitated several community-based research projects and door-to-door health interventions in a densely populated, non-notified slum in Mumbai, India. The coding system doubled as walking directions, which allowed rapid identification of individual in their households by a research team of youth from Mumbai, many of whom live in the slum community itself. For the cost of a few hours of training and one computer for data management, LBHCS allowed a household census and reliable household re-identification for over one year. Re-enumeration of all households was performed for a new study in just four days by ten people.

By combining a LBHCS with GIS, two people mapped several thousand households in just a few days, which allowed spatial analysis of detailed household survey data. Mapping at this scale by other methods would not have been possible, or it would have been prohibitively time-intensive and expensive. By generating a few additional spatial datasets, PUKAR researchers produced base maps of community infrastructure that added leverage in several important meetings between Kaula Bandar residents and city officials to extend city services.

Although Kaula Bandar is geographically small, these methods should easily scale to slums with larger populations covering larger geographic areas. The simplicity of the coding system means rapid training for any sized research or outreach team, including teams composed of members who are young or who have limited technology skills. LBHCS is extremely effective for following the same households over weeks and months because the codes double as clear walking directions. These methods are particularly amenable to high-density slums in which new development occurs on the periphery of the community, and new dwellings are not frequently added between existing dwellings. LBHCS is an efficient, easy-to-learn, cost-effective, scalable approach to household enumeration and re-identification in densely populated settings.
